# Case report on ear numbness following deep brain stimulation implantation

**DOI:** 10.1016/j.ijscr.2022.106773

**Published:** 2022-01-15

**Authors:** Agus Turchan, Achmad Fahmi, Takaomi Taira, Heri Subianto, Asra Al Fauzi, Resi Prastikarunia

**Affiliations:** a Faculty of Medicine, Universitas Airlangga, Dr. Soetomo General Academic Hospital, Surabaya, Indonesia; b Department of Neurosurgery, Tokyo Women's Medical University, Tokyo, Japan

**Keywords:** Ear numbness, Deep brain stimulation, Parkinson's disease

## Abstract

**Introduction and importance:**

Deep brain stimulation (DBS) implantation is a neurosurgical procedure in which electrodes are implanted in the brain. Complications that may occur include wound infection, issues with the DBS hardware, and others. This case report presents a patient who suffered ear numbness following DBS implantation.

**Case presentation:**

A 50-year-old man presented with resting tremors in both hands. He reported that his handwriting had worsened and his movements had slowed. Physical examination revealed rigidity and postural instability. The patient was diagnosed with Parkinson's disease and a bilateral subthalamic nucleus DBS implantation was scheduled combined with the patient's medication all this time. Patient's symptoms showed improvement after the procedure. However, the patient complained of ear numbness and occasional pain in the area around his ear. We observed the patient over the next 3 months and the symptoms eventually resolved without any medication and intervention.

**Clinical discussion:**

Ear numbness is a rare complication that occasionally occurs after DBS implantation. This complication occurs because the tunneling track's proximity to the great auricular nerve and the lesser occipital nerve can result in accidental damage to either one or both of these nerves during subcutaneous tunneling.

**Conclusion:**

We suggest a simple procedure to avoid neural injury while maintaining the course of the tunneling in which the tunnel is created below the periosteum rather than at the subcutaneous level.

## Highlights

• Ear numbness is a rare complication after DBS.
• The likely cause is neural damage during subcutaneous tunneling.
• In DBS implantation, tunneling below the periosteum can prevent this complication.

## 1 Introduction

Deep brain stimulation (DBS) implantation is a neurosurgical procedure in which electrodes are implanted in the brain [1]. Since its introduction, this procedure has become widely used for a variety of conditions [1]. Despite its proven efficacy to treat movement disorders like Parkinson's disease (PD), this procedure has complications risk such as wound infection, issues with the DBS hardware, and others [1]. This case report presents a patient with PD who suffered ear numbness following DBS implantation, the relationship between ear numbness and DBS and discusses how to prevent such a complication. This work has been reported in line with the SCARE guideline [2]. This work has been registered at http://www.researchregistry.com (researchregistry7411).

## 2 Presentation of case

A 50-year-old man presented with tremors in both hands, which were more apparent when resting. The patient also reported that his handwriting had worsened and his movements had slowed. Physical examination revealed rigidity and bradykinesia. The patient was diagnosed with PD and we performed a bilateral subthalamic nucleus (STN) DBS implantation.

The procedure was planned using preoperative magnetic resonance imaging (MRI) and preoperative stereotactic computerized tomography (CT) to identify the location of the STN. A skin incision was made based on the computer-generated coordinate. The DBS electrode was inserted through a double burr hole and a cable was subcutaneously tunneled behind the ear. Patient's symptoms including resting tremor, rigidity and bradykinesia showed improvement after the procedure. However, the patient complained of numbness and occasional pain in the area around his ear. This caused sufficient discomfort to affect the patient's subjective quality of life.

The patient reported that the pain could be elicited by relatively trivial stimuli, such as putting on an earphone or putting a phone to his ear. The numbness, on the other hand, was present at all times. We suspected that these symptoms were due to injury to the greater auricular nerve and/or the lesser occipital nerve, both of which run close to the subcutaneous DBS tunneling path. We observed the patients over the subsequent 3 months and the symptoms eventually resolved without medical intervention.

## 3 Discussion

There are a number of complications associated with DBS implantation [1]. A study of 728 patients who underwent the procedure reported that intraoperatively, intracranial hemorrhage was found to be the most common intraoperative complication [1]. Postoperative complications that have been reported include transient dysphasia, transient clumsiness, rebound tremor, infection, seizure, and hardware issues [3]. Problems that can occur with DBS hardware include electrode failure, extension wire failure, pulse generator malfunction, and pain over pulse generator area [4].

**Fig. 1:**
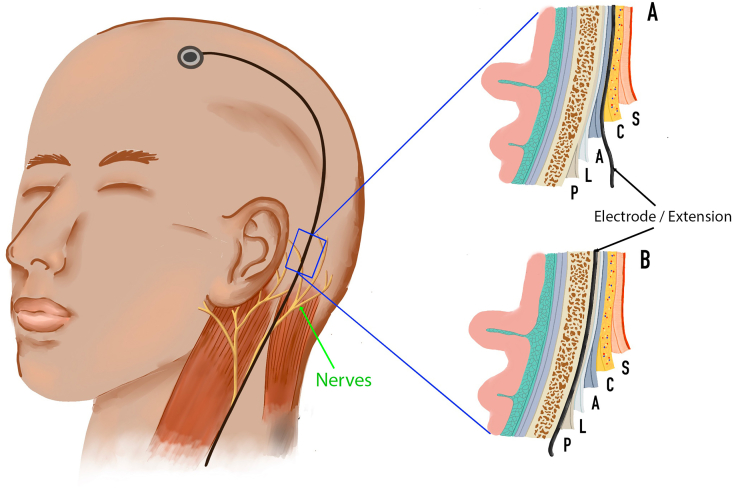
Comparison of tunneling layers that may be used during implantation of deep brain stimulation (DBS) devices. A: Location of a DBS cable that tunnels through the subcutaneous tissue. B: Location of a DBS cable that tunnels below the periosteum. P = periosteum, L = loose connective tissue, A = aponeurosis, C = subcutaneous, S = skin.

Ear numbness after DBS is rare [5]. However, given the tunneling track's proximity to the great auricular nerve (GAN) and the lesser occipital nerve (LON), we hypothesize that postoperative ear discomfort is caused by injury to one or both of these nerves during subcutaneous tunneling (Fig. 1A). In most cases, this issue resolves itself without intervention within about 3 months to 1 year but some patients have reported persistent symptoms lasting up to 5 years after DBS implantation [5]. Fortunately, the symptoms resolved spontaneously in the present case.

Injury to the GAN may occur during otorhinolaryngological surgeries, and head and neck surgical procedures, such as parotidectomy, neck dissection, rhytidectomy, and excision of lateral neck masses [6]. The GAN is a sensory nerve that arises from the C2 and C3 spinal nerves [6]. Although it is protected by the sternocleidomastoid muscle, it emerges superficially on its path toward the parotid gland [7]. The GAN then divides into an anterior branch, which innervates the skin overlying the parotid gland, and a posterior branch, which innervates the skin over the mastoid, the posteroinferior surface of the auricle, the lobule, and the concha [6].

Research has produced measurements to assist surgeons operating in this area, helping them to avoid injuring the GAN [[7], [8], [9], [10]]. McKinney et al. estimate that the main trunk of the GAN crosses the sternocleidomastoid at a point 6.5 cm below the external auditory canal [8]. The posterior branch of the GAN is especially vulnerable in neurosurgical procedures such as ventriculoperitoneal (VP) shunt or DBS implantation, which require tunneling around the area it innervates. The posterior branch is approximated to be 1.5 cm behind the ear lobule insertion [9]. The point at which the GAN divides into its posterior and anterior branches is approximated to be 29.1 mm below the tip of the mastoid process parallel to the anterior border of the sternocleidomastoid muscle [10].

The point 1.5 cm posterior to the lobule insertion might be a useful marker when placing the skin incision for tunneling in DBS implantation. However, utilization of all of the aforementioned measurements to determine nerve placement may be too time-consuming during DBS implantation. We, therefore, propose that a simple means of avoiding injury to nerves without altering the tunneling course would be to tunnel below the periosteum instead of at the subcutaneous level (Fig. 1B).

## 4 Conclusion

Tunneling below the periosteum instead of at the subcutaneous level is a simple change of procedure that avoids neural injury during DBS implantation without altering the tunneling course. Further research is required to prevent ear numbness after DBS implantation.

## Consent

Written informed consent was obtained from the patient for publication of this case report and accompanying images. A copy of the written consent is available for review by the Editor-in-Chief of this journal on request.

## Provenance and peer review

Not commissioned, externally peer-reviewed.

## Ethical approval

All of the procedures performed in this study involving human participants were in accordance with the ethical standards of the institutional research committee.

## Funding

This article received no specific funding from any funding agency in the public, commercial, or non-profit sectors.

## Guarantor

Achmad Fahmi, MD, PhD.

## Research registration number

1. Name of the registry: http://www.researchregistry.com
2. Unique identifying number or registration ID: researchregistry7411
3. Hyperlink to your specific registration (must be publicly accessible and will be checked): https://researchregistry.knack.com/research-registry#home/registrationdetails/61a4a0a2313372001ec99e47/.

## CRediT authorship contribution statement

Agus Turchan, MD, PhD: study concept or design, data analysis or interpretation, writing the paper
Achmad Fahmi, MD, PhD: study concept, data collection, writing paper, critical revised article and supervising
Prof. Takaomi Taira, MD, PhD: critical revised article and supervising
Heri Subianto, MD: study concept or design, writing paper
Asra Al Fauzi, MD, PhD: study concept or design
Resi Prastikarunia, MD: revised English writing.

## Declaration of competing interest

None.

## Abbreviations

DBS: Deep brain stimulation
CT: Computerized tomography
MRI: Magnetic resonance imaging
GAN: Great auricular nerve
LON: Lesser occipital nerve
VP Shunt: Ventriculoperitoneal shunt
SCALP: Skin, subCutaneus, Aponeurosis, Loose connective tissue, Periosteum
